# Supplementary material for: Vanadium-Substituted Dawson-Type Polyoxometalate–TiO2 Nanowire Composite Film as Advanced Cathode Material for Bifunctional Electrochromic Energy-Storage Devices
Source: Molecules. 2022 Jul 4;27(13):4291. doi: 10.3390/molecules27134291 (PMC9268091; doi:10.3390/molecules27134291)
Supplement: Supplementary file 1 [file molecules-27-04291-s001.zip › molecules-1778779-supplementary.pdf]

Supplement files

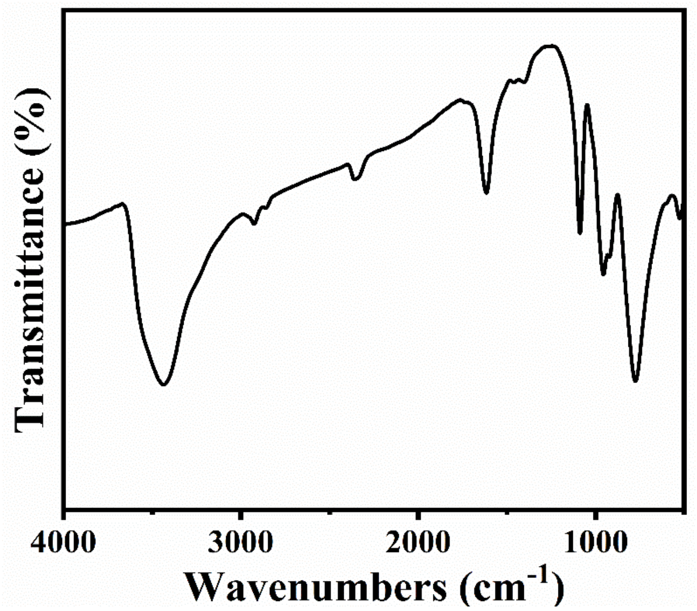

Figure S1. The IR spectra of $K_7[P_2W_{17}VO_{62}]\cdot18H_2O$.

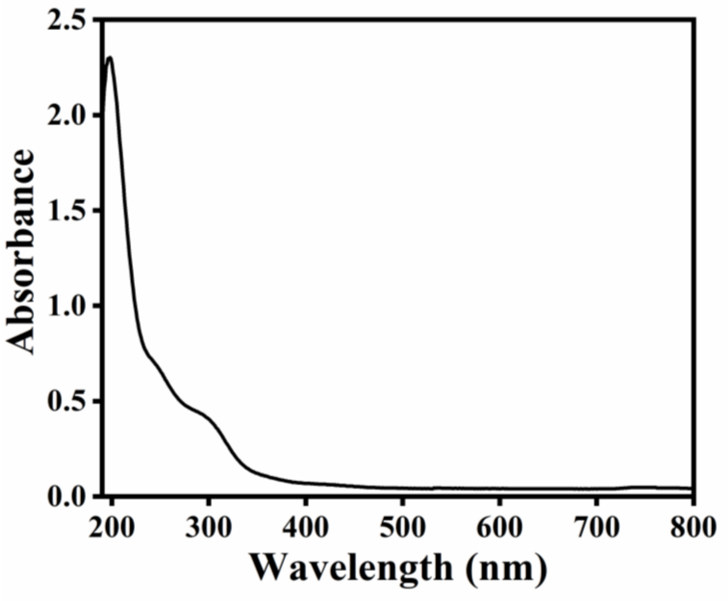

Figure S2. The UV-vis spectra of $K_7[P_2W_{17}VO_{62}]\cdot18H_2O$.

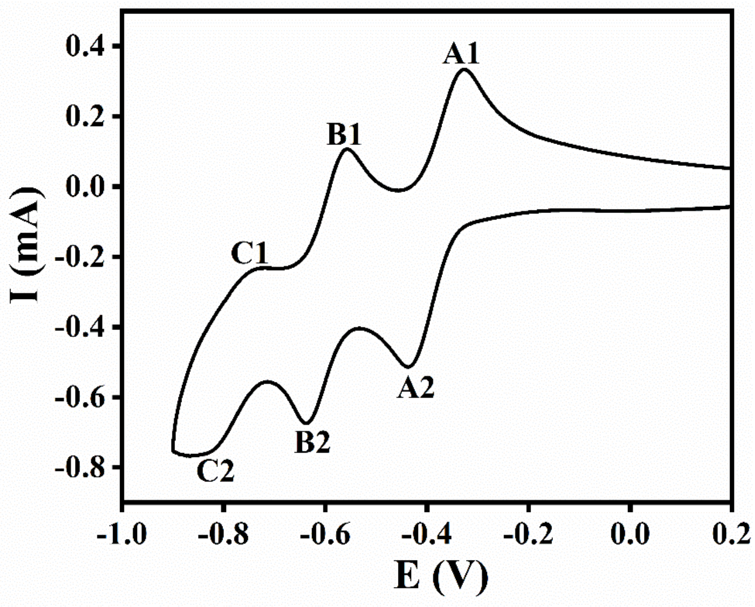

Figure S3. CV curve of $K_7[P_2W_{17}VO_{62}]\cdot 18H_2O$ in HOAc-NaOAc solution (pH=3.5).

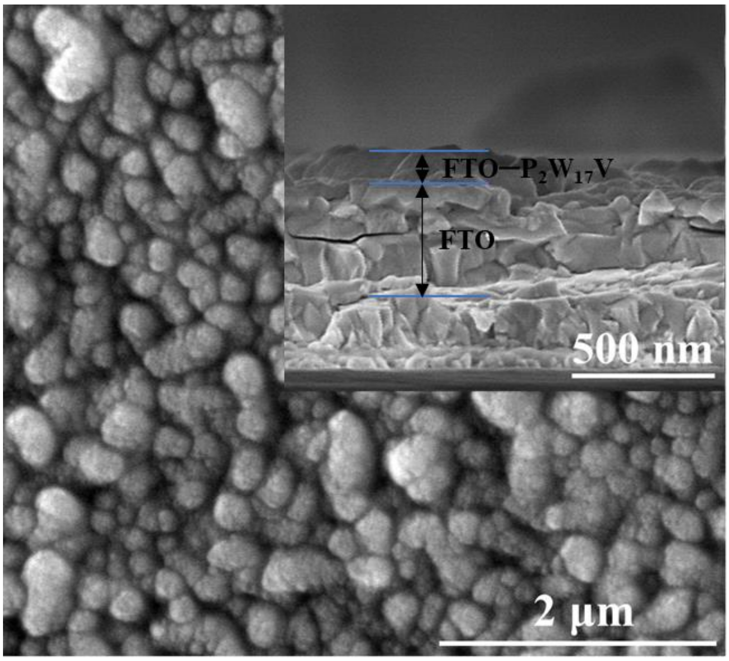

Figure S4. The SEM images of FTO–$P_2W_{17}V$ (inset: the cross-sectional images of prepared films).

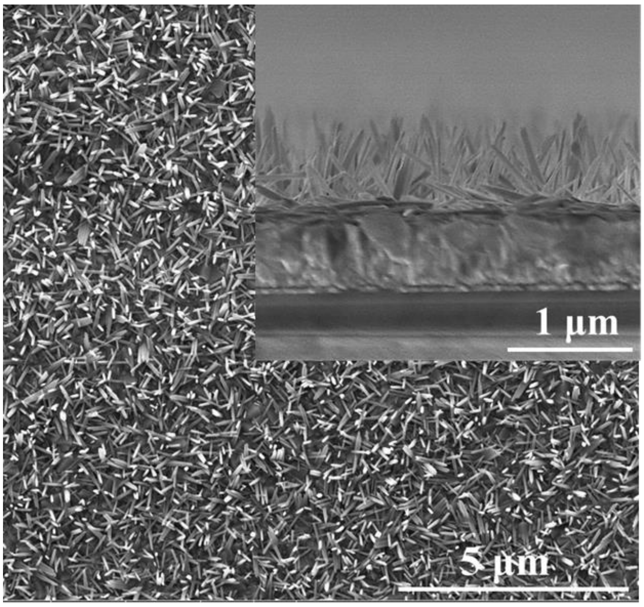

Figure S5. The SEM images of TiO_2 NW (inset: the cross-sectional images of prepared films).

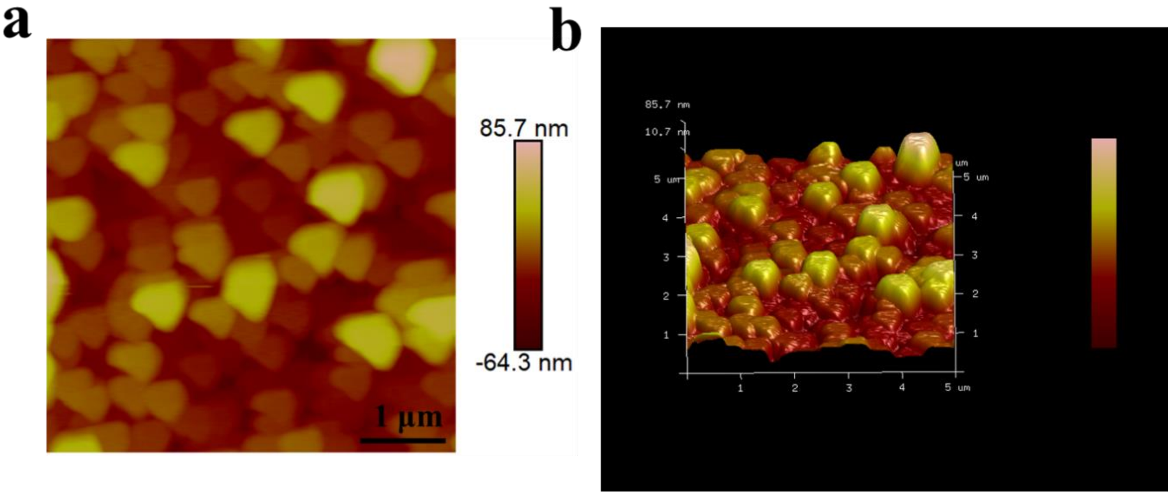

Figure S6. 2D AFM images of (a) FTO–$P_2W_{17}V$ and 3D AFM images of (b) FTO–$P_2W_{17}V$.

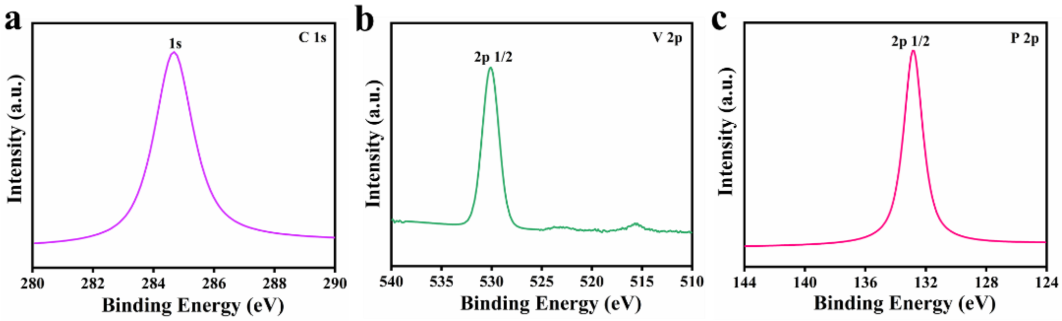

Figure S7. High-resolution XPS spectra for C1s (a), V2p (b) and P2p (c) of FTO–P_2W_{17} film.

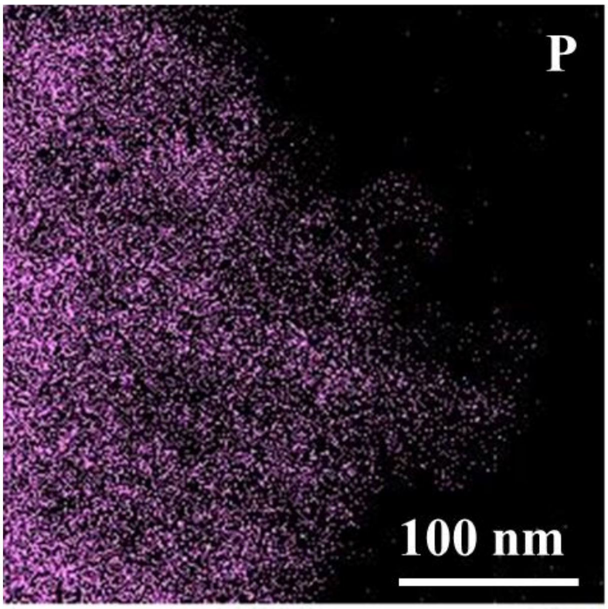

Figure S8. EDS elemental mapping patterns of P in the NW–$P_2W_{17}V$ film.
